# Return to Work or Not: The Paths to Psychiatric Disability and Back

**DOI:** 10.1007/s10926-025-10312-4

**Published:** 2025-08-11

**Authors:** Päivi Rissanen, Sami Pirkola, Turkka Näppilä, Tino Karolaakso, Helena Leppänen, Sari Fröjd, Reija Autio

**Affiliations:** 1 Faculty of Social Sciences (Unit of Health Sciences), Tampere University, Arvo Ylpön Katu 34, 33520 Tampere, Finland; 2 Department of Psychiatry, The Wellbeing Services County of Pirkanmaa, Tampere, Finland; 3 Tampere University Library, Tampere University, Tampere, Finland; 4 Faculty of Medicine and Health Technology, Tampere University, Tampere, Finland

**Keywords:** Mental disorder, Disability pension, Work disability, Unemployment, Sequence analysis

## Abstract

**Objectives:**

Mental health-related work disability has increased in Europe, despite efforts to promote individuals´ work ability. We examined individuals´ occupational status before and after a psychiatric disability pension (DP).

**Methods:**

The study comprises individuals granted a DP for the first time between 2010 and 2012 in Finland (*N* = 18,373). We used modern methods to cluster the sequences of individuals´ occupational status before and after temporary (*n* = 8615) or permanent (*n* = 9758) psychiatric DP. We compared socioeconomic, illness and health care system-related factors between nine groups, formed by sequence analysis utilizing multinominal regression analysis.

**Results:**

The analysis identified typical groups of temporary pensioners: after steady working careers, periods of unemployment and from mixed states. Severity of health and mental health problems, socioeconomic and occupational status, pension system-related factors as well as treatment or rehabilitation varied between the groups. Individuals with temporary DPs (tDP) appeared mainly to either remain disabled (74% of the study tDP) or return to the same status they had before disability: to work (17%) or unemployment (8%). A steady working career, high education and received psychotherapy and rehabilitation all promoted returning to work. Among young adults, severity of the illness and lack of occupational education were risk factors for long-term disability.

**Conclusions:**

Among those with tDP, circles of disadvantage may exist. They relate to unemployment, poor mental and somatic heath, low education, poverty, and failure of rehabilitative efforts. Especially young adults with severe mental disorders require not only rehabilitation but also educational support.

**Supplementary Information:**

The online version contains supplementary material available at 10.1007/s10926-025-10312-4.

## Return to Work or Not: Paths to Psychiatric Disability and Away from it

Mental disorders are one of the main reasons for disability pensioning [1], especially among young adults [2]. Psychiatric occupational disability is a common health-, economic- and social policy challenge in several European countries [1]. There is a clear political and economic emphasis on encouraging people to continue working and avoiding a permanent exit from working life [3]. However, only a few European countries have tried to address the impacts of longer-term absence from the labour market on sickness benefits, the main purpose of which is to avoid permanent exit from the labour market [4].

People with mental disorders are assigned to a disadvantaged group or a specific risk group in the labour market [3, 4]. Especially people with severe mental disorders have a high probability of never entering the active labour market, of dropping out of it, or of becoming long-term unemployed [5]. Based on earlier studies, the association between unemployment and mental disorders may be bidirectional [6]. There is evidence that poor mental health may lead to unemployment, which increase the risk of poor health [7–9], and that there is a reciprocal relationship between them [9].

Previous studies have consistently found associations between sociodemographic factors and the prevalence and presentation of mental disorders as well as in disability pensioning and service utilization. Both mental disorders and disability pensioning are more prevalent among individuals with low SES. [10–13]. Based on a systematic literature review, women were granted disability pensions (DPs) due to mental disorders more frequently than men, and affective disorders were the most common diagnoses [10]. Additionally, the prevalence of depression and anxiety is higher among women, whereas substance use disorders and antisocial behaviours are more common among men [1]. One Finnish study suggested that most people with a DP due to major depression had comorbid mental or physical disorders which contributed to their disability However, the relationship between SES and the utilization of health care services remains unclear [11, 14].

National differences exist in ways to offer treatment and rehabilitation as well as to promote individuals’ work ability [2]. These differences may influence equity in access to, and the quality of, mental health treatment and rehabilitation [15]. For example, financial cost can affect individuals’ service utilization [16, 17]. In several countries, the Individual Placement and Support (IPS) model is in use. This model involves employment specialists collaborating closely with mental health treatment teams to deliver tailored support for employment [18].

In addition, entitlement to sick leave and sickness benefit schemes varies remarkably between countries [4]. However, in all EU member countries, employees have the right to sick leave and sickness benefit substituting loss of income during absence from work for sickness. The sickness and disability benefit systems are usually constructed in such a way that long-term sickness absence precedes permanent DP [19, 20]. Consequently, there are transition processes between benefits from long-term sickness to permanent disability [4]. In Finland, sickness allowance is granted for disabilities lasting less than 300 days. If a chance exists for work ability to be restored, temporary DP can be granted. This allows re-evaluation of the subject’s work ability before possible permanent DP and an early exit from working life [4, 19]. The DP can be granted to individuals aged 17–63 who are unable to continue in their current position due to illness, injury or handicap [2]. Assessment of the right to DP is always based on a medical statement of health and the individual’s ability to obtain an income through work, while their education, previous work, age and place of residence are all considered. DPs may be paid either through earnings-related schemes or the national pension scheme, which guarantees a minimum level of pension and earning.

Based on previous research, return to work (RTW) after a long-term sickness absence due to mental disorders is quite uncommon [5, 21]. Possible predictors of RTW are not only related to the severity of mental disorders but also to sociodemographic, psychological, treatment, and work related factors [14]*.* Numerous studies have indicated that specifically, women, young adults, and highly educated exhibit higher rates of returning to work [2, 7, 8, 22]. This tendency is also observed in individuals without concurrent substance abuse issues or significant psychological symptoms during their childhood [23, 24].

Previous psychotherapy and rehabilitation appeared to increase the probability of RTW in several Finnish studies [21, 25, 26], but some studies have found no clear correlation between counselling, exercise, medical rehabilitation or RTW programmes and RTW [27–29].

Unemployment [20, 30], the threat of unemployment [31] as well as temporary employment [32] before work disability were associated with prolonged RTW or no RTW in several studies. Accordingly, people who were employed before work disability [21, 25], had a sustained employment history [23, 24] or had an ongoing employment contract throughout the disability benefit period [33], had a higher probability of RTW. Moreover, environmental factors such as job demands, supervisory and co-worker support were related to RTW [14].

## Gap and Aims

Psychiatric occupational disability has been a common health-, economic- and social policy challenge in several European countries in recent decades [1]. Moreover, mental health-related work disability has increased, despite efforts to promote individuals´ work ability. Earlier studies have investigated factors related to disability pensioning, unemployment and RTW after absence from work due to mental disorders, but less attention has been paid to preceding long-term absence from work or multiple paths and transitions related to the pensioning processes. This study aims to characterize the overall process and temporal sequences of premature disability pensioning due to mental disorders and retirees’ occupational situations before and after early retirement.

## Methods

This study is part of the research project RETIRE—*“*Paths of disability and recovery: Determinants of mental health-based disability pensioning in the light of system-level- and register-based information” [5, 11–13, 26, 34, 35].

## Study Population and Variables

The study population contains all individuals in Finland who were granted a DP (*N* = 18,373) due to mental disorder (ICD10: F10-F69, F80-F99) for the first time between 2010 and 2012. We limited the population by excluding those granted a DP prior to 1st January 2010 and included in the study only those retirees who could be followed up followed up for 3 years from the beginning of their DP, to obtain a reliable view of the long-term outcomes of pensioning.

The data were obtained from several nationwide registers: the Finnish Centre for Pensions (FCP), the National Social Insurance Institution (SII), the Finnish Hospital Discharge Register (FHDR) and Statistics Finland (SF). Sociodemographic and socioeconomic data were measured at the beginning of the pension from registers of SF. Education level was categorized based on the duration of education and occupational status into seven categories in accordance with SF’s classification. Household disposable income was calculated based on OECD’s consumption unit and classified to five quantiles. The diagnoses for granting the DP were classified as F2*(F20-F29) Schizophrenia, schizotypal and delusional disorders, F3*(F30-F39) Affective disorders, F4*(F40-F48) Neurotic disorders and other (F1*, F5*-F6*, F8*-F9*). Comorbidity was categorized based on DP decisions as: no comorbidity, comorbid psychiatric, somatic, and substance abuse diagnosis. Regarding the service-related factors, data on previous psychotherapy and different forms of rehabilitation were recorded up to 5 years before being granted DP and classified as ‘yes’ or ‘no’. Data were collected from the registers of the SII and the FCP. Psychiatric inpatient treatment data were measured by five years before the granted DP and categorized as: no treatment; 1–14; 15–30; and over 31 days in hospital. Involuntary treatment data were measured by five years before the granted DP and categorized as ‘yes’ or ‘no’. Data were collected from the registers of the FHDR. As a pension system-related factor we used the number of rejected DP applications before the first granted DP. This was categorized as 0; 1 or 2; 3 and over was rejected. Data were collected by five years before the granted DP from the registers of the FCP and the SII.

Analysis of the data consisted of three main parts: (1) cluster analysis of the state sequences to characterize typical paths to and after temporary and permanent DP; based on that, (2) a description of the nine groups formed in part 1; (3) multinominal regression analysis for detecting the risk factors in each group.

Labour market status up to 5 years before and 3 years after DP was analyzed with sequence analysis and clustering, the methodology is described previously [5]. Unlike in our previous analyses, we here present separated sequence analysis of the individuals’ states before and after temporary and permanent DPs, in order to identify typical paths to and after DP. Each month, an individual was assigned to a state: permanent psychiatric DP, temporary psychiatric DP, non-psychiatric permanent DP, non-psychiatric temporary DP, sickness allowance, student, unemployed, or work. When multiple states were found simultaneously, they were combined and labeled by the dominant state, as in [5]. The clustering algorithm computes the distances between individuals’ states and identifies individuals with similar state patterns more accurately than traditional statistical methods, as it can detect pattern similarity even though the states vary monthly and are not identical between individuals. The individuals were clustered, and state distributions were computed over time based on their individual sequences of states. The states were clustered using an agglomerative hierarchical clustering algorithm with optimal matching with constant distances and Ward’s linkage method.

This clustering analysis was performed separately for the temporary DP and permanent DP groups, and the algorithm identified six clusters for the temporary DP and three clusters for the permanent DP group. We have illustrated the 500 most common sequences of each cluster to show the typical paths for each cluster. This limited set of sequences captures a substantial proportion of observations and displaying all is infeasible due to the extensive dataset. The analysis was conducted with R using the TraMinerR, cluster, ggplot2, packages. [36–38].

We then investigated whether the sociodemographic, health and system-related factors were connected to the membership of groups in order to identify factors related to returning to work. Finally, for the associations between explanatory variables and groups, we used multinomial logistic regression analysis. Groups with permanent DPs were reference groups in regression analysis, because the subjects had permanent work disability and high probability to remain in DP without dispersions in paths of states. We analysed the association between each variable with both the univariable and multivariable model and computed the odds ratios (ORs) for the outcomes. To identify the best model for the data, we entered into the model the variables with p < 0.05 at least for one of the comparisons and evaluated the goodness of the models with the Nagelkerke Pseudo R^2^ and the deviance tests. Regression analyses were performed using SPSS software (version 27).

## Results

### Descriptive Information and the Clusters Identified

We identified 18,373 individuals who were granted temporary (*n* = 8615) or permanent (*n* = 9758) DP due to mental disorder between 2010 and 2012. Their mean age was 42 years (SD 12.8) and 53% were female. The most common psychiatric disability diagnoses were affective disorders (65%), followed by psychotic disorders (18%). Based on the primary analysis, we found three typical main paths to both temporary and permanent DPs: (1) after steady working careers, (2) after long-term unemployment or repeated periods of unemployment and (3) from mixed states (individuals were studying, working or being unemployed before DP).

**Fig. 1:**
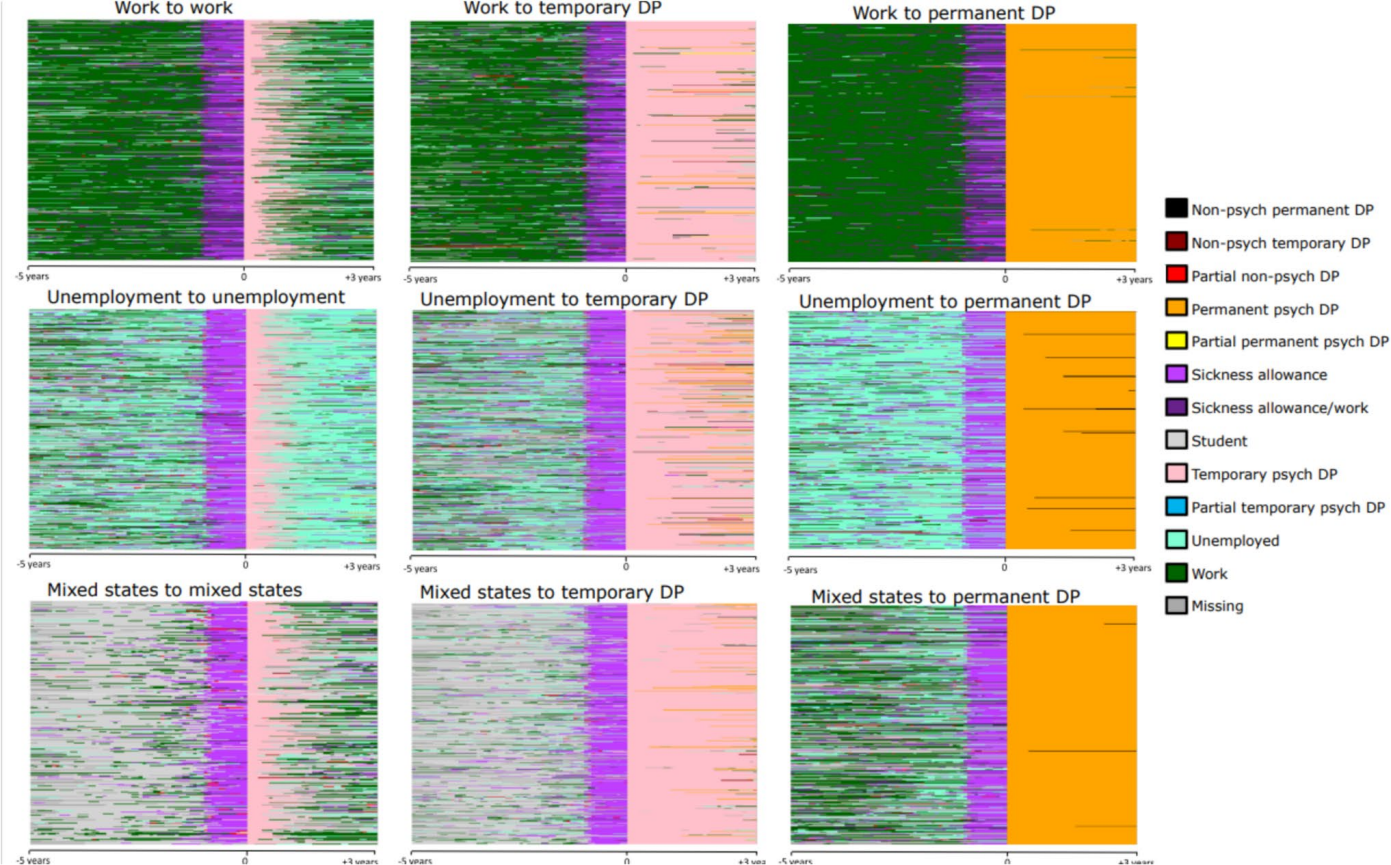
Nine representative paths to and after a temporary and a permanent disability pension, acquired by clustering of individual state sequences. The figure shows 500 of the most common sequence trends prior to and after pensioning within each group

After temporary DP, individuals mainly returned to work, unemployment or studying or remained in DP, whereas the vast majority of permanently retired remained in DP (98%). Hence, we clustered separately the individuals with permanently and temporary DP. The clustering algortihm identified nine groups based on individuals’ paths to and after DP; six clusters for temporary DP and three clusters for permanent DP (Fig. 1 and Supplementary material 1) and we named them after the main paths of individuals of each group. As a result of these cluster analyses, the individuals with a working career (*n* = 7728) background clustered to the ‘work to work ‘, ‘work to temporary disability ‘ and ‘work to permanent disability ‘ groups. Individuals with unemployment periods before DP (*n* = 5661) were assigned to groups labelled ‘unemployment to unemployment ‘, ‘unemployment to temporary disability ‘and’ unemployment to permanent disability ‘. Individuals with mixed states (*n* = 4948) were at work, unemployed or studying before DP and were called ‘mixed states to mixed states ‘, ‘mixed states to temporary disability ‘ and ‘mixed states to permanent disability ‘ groups. Figure 1 shows 500 of the most common sequence trends prior to and after pensioning within each group. In addition, the states and transitions in each group, formed by the sequence analysis, can be seen in Supplementary material 1. Sociodemographic, mental health, health and system-related factors were highly significantly associated with group membership (*p* < 0.001) (Table 1).

**Table 1:** Description of sociodemographic, socio-economic, health, health care and pension system-related factors of people granted temporary or permanent disability pension for mental reasons between 2010 and 2012

	Steady working careers before disability (*n* = 7728) 42.1%			Long-term unemployed or repeated periods of unemployment before disability (*n* = 5661) 30.7%			Mixed states before disability (*n* = 4948) 27.2%			Total (*N* = 18,373)
	Work to work-group	Work to temporary disability -group	Work to permanent disability-group	Unemployment to unemployment -group	Unemployment to temporary disability-group	Unemployment to permanent disability-group	Mixed states to mixed states-group	Mixed states to temporary disability -group	Mixed states to permanent disability-group	Total
	(*n* = 1445) 7.9%	(*n* = 2349) 12.8%	(*n* = 3934) 21.4%	(*n* = 486) 2.6%	(*n* = 2100) 11.4%	(*n* = 3075) 16.7%	(*n* = 323) 1.8%	(*n* = 1912) 10.4%	(*n* = 2749) 15.0%	(*N* = 18,373) 100%
Gender										
Male	36.3	36.4	44.1	40.5	46.3	56.1	39.4	49.1	53.7	46.6
Female	63.7	63.6	55.9	59.5	53.7	43.9	60.6	50.9	46.3	53.4
Age										
Mean	40.2	38.0	53.4	36.5	35.8	49.5	22.1	22.9	47.1	42.1
Std	9.6	9.9	6.9	10.5	9.4	9.2	4.8	5.3	11.4	12.8
Education level										
Basic level	15.4	18.7	20.9	38.9	41.0	36.5	46.4	55.3	35.1	31.7
Upper secondary	52.0	54.2	47.0	47.1	46.8	51.2	48.6	40.3	46.2	48.2
Tertiary	24.5	20.9	24.3	11.9	9.7	10.0	2.8	3.6	15.0	15.6
High	8.1	6.2	7.7	2.1	2.6	2.4	2.2	0.8	3.7	4.5
Family situation										
Single	32.9	36.3	32.0	49.8	49.9	63.5	42.9	55.2	51.1	45.9
Couple	21.4	20.4	42.2	17.3	13.8	18.3	18.9	10.2	24.3	23.5
Single + children	14.8	13.6	6.4	13.8	13.5	7.2	12.7	11.2	7.6	9.9
Couple + children	30.9	29.7	19.3	19.1	22.9	11.0	25.5	23.4	17.0	20.7
Disposable income of household euros/year										
Lowest	17.6	23.7	10.6	61.5	61.9	71.5	49.8	52.7	45.4	40.2
Middle-lower	29.3	29.0	20.6	24.4	23.0	19.7	18.8	21.4	26.0	23.4
Middle	24.0	21.6	23.7	7.2	9.1	5.3	14.7	13.4	14.7	15.8
Middle-higher	16.8	14.9	22.6	4.2	3.9	2.3	10.9	8.3	8.4	11.4
Highest	12.3	10.9	22.5	2.7	2.1	1.2	5.8	4.2	5.4	9.2
Occupational status										
Self-employed	8.3	7.6	9.3	2.1	3.1	0.9	2.8	2.0	5.8	5.3
Upper non-manual employees (administrative, managerial, professional and related occupations)	13.1	9.3	15.6	1.6	3.7	1.6	5.6	4.0	4.9	7.5
Lower non-manual employees (administrative and clerical occupations)	34.0	31.2	35.8	8.4	8.6	5.1	8.7	7.5	10.8	18.9
Manual workers	22.8	26.7	28.3	12.8	10.6	7.0	6.5	7.7	12.5	16.8
Student	7.7	9.0	1.1	15.0	11.7	2.9	59.4	50.2	8.5	11.8
Unemployed	4.0	4.7	3.7	16.0	16.0	33.1	2.8	6.4	17.0	12.8
Unknown	10.1	11.5	6.2	44.0	46.3	49.5	14.2	22.1	40.5	26.9
Psychiatric diagnosis										
F2 Psychotic disorders	9.4	15.0	10.1	14.0	23.9	17.9	15.8	38.4	20.9	18.3
F3 Affective disorder	82.8	75.3	80.4	69.1	58.8	47.9	59.1	40.7	57.0	63.7
F4 Neurotic disorders	5.5	6.2	4.9	9.9	10.0	7.7	15.2	7.4	4.7	6.7
Other	2.4	3.5	4.7	7.0	7.3	26.5	9.9	13.4	17.4	11.3
Comorbid disorder										
No	51.8	40.4	39.0	38.1	33.7	25.9	39.0	42.2	36.5	37.3
Other psychiatric	37.2	47.6	35.2	52.1	57.1	47.7	56.3	54.4	43.8	45.6
Somatic	10.9	12.0	25.8	9.9	9.2	26.4	4.6	3.5	19.7	17.0
Comorbid substance abuse disorder	3.4	3.0	2.1	6.6	6.9	6.3	0.1	3.6	5.4	4.7
Previous psychotherapy										
No	74.4	72.8	88.1	87.7	83.7	97.4	66.6	75.6	93.6	85.2
Yes	25.6	27.2	11.9	12.3	16.3	2.6	33.4	24.4	6.4	14.8
Previous rehabilitation										
No	53.6	56.5	77.5	67.1	64.6	80.3	61.3	55.9	74.9	68.7
Yes	46.4	43.5	22.5	32.9	35.4	19.7	38.7	44.1	25.1	31.3
Hospital treatment										
No	7.9	6.3	9.1	8.6	8.0	10.1	17.6	10.6	8.5	8.9
1–14 days	40.0	32.5	44.1	36.4	26.9	35.2	27.6	19.1	33.2	34.1
15–30 days	15.8	15.1	15.5	14.4	13.2	14.5	5.3	8.5	14.5	14.0
Over 31 days	36.3	46.1	31.3	40.5	51.9	40.2	49.5	61.8	43.8	43.1
Involuntary treatment										
No	87.3	81.8	88.5	84.4	74.7	84.0	78.3	64.9	80.0	81.2
Yes	12.7	18.2	11.5	15.6	25.3	16.0	21.7	35.1	20.0	18.8
Number of rejected disability pension applications										
0	42.5	49.3	59.7	33.5	42.4	51.9	51.7	62.6	53.6	50.9
1–2	45.1	42.4	30.4	43.5	44.4	20.9	41.7	35.7	28.0	38.5
3-	12.4	8.3	9.9	23.0	13.2	27.2	6.6	1.8	18.4	10.6

Columns indicate the groups identified by the cluster analysis, i.e., people belonging to the work to work, work to temporary disability, work to permanent disability, unemployment to unemployment, unemployment to temporary disability, unemployment to permanent disability, mixed states to mixed states, mixed states to temporary disability and mixed states to permanent disability -groups, %

### Individuals with Steady Working Careers

Individuals who transferred to DPs after long and steady working careers were mainly employed up to the granting of DP, after 300 days sick leave from work (Fig. 1 and Supplementary material 1). Common features to these groups were high proportions of individuals with affective disorders and high education and income levels.

Individuals who returned to work after DP comprised ‘*the work to work -group’* (*n* = 1445). This group had the highest proportion of women (64%), as well as of individuals with high education (8%) and living with partner and children (31%). Furthermore, individuals most frequently had affective disorders (83%), as well as previous rehabilitation (46%) and short inpatient treatments (40%) (Table 1). They were at work on average for 13.9 months during the follow-up time and 82% of them were at work for at least one month; a minority had unemployment periods or started studies during follow-up.

The *‘Work to temporary disability’* -group (n = 2,349) was composed of individuals who mainly remained in temporary DP during the follow-up period. Although their demographics were quite similar to those of the previous group, they were younger, had lower education and income levels, were more frequently manual workers and more often had a psychotic diagnosis. However, the clearest difference was in the length of inpatient treatment. Individuals in this group more frequently had long psychiatric hospital treatments and less frequently short treatments compared to individuals who returned to work (Table 1).

Finally, there were individuals who were granted permanent DP immediately. The ‘*Work to permanent disability’-group* (*n* = 3934) was the oldest, economically most advantaged group and individuals lived most frequently with partners. The distinctive feature of this group was the high number of individuals with comorbid somatic disorder (frequently diseases of the musculoskeletal system and connective tissue) and the low proportion of individuals receiving psychotherapy and rehabilitation (Table 1).

### Unemployment as a Background Before Disability

Individuals who were either long-term unemployed or had repeated periods of unemployment transferred to DP via sick leave when unemployed or at work. Characteristic to these groups were low education and income levels as well as having several (≥ 3) rejected DP applications before granting of DP. Furthermore, they more frequently had substance-related diagnosis as a comorbid psychiatric diagnosis than individuals with a steady working career background.

Individuals who returned mainly to unemployment after being in DP formed the ‘*unemployment to unemployment’ -group* (*n* = 486) (Fig. 1). A minority of them had some working days during the follow-up period, but only 1.5 months on average. Individuals who remained in DP during follow-up comprised the ‘*unemployment to temporary disability’ -group* (*n* = 2100). The main differences in demographics were seen in the distributions of diagnosis and treatment. Individuals in the latter group not only more frequently had a psychotic diagnosis but also long inpatient treatments (Table 1).

Individuals who transferred directly to permanent DP, i.e., the ‘*unemployment to permanent disability’ -group* (*n* = 3075) differed from other retirees in having the highest proportion of males and of individuals living alone. Furthermore, it was the economically least advantaged group, with the highest proportion of individuals whose SES was unknown (50%) before disability (Table 1). This group also most frequently had individuals with brain-organic disorders (F0*) (3.7%) and a substance-related diagnosis (14%), which was often a comorbid psychiatric diagnosis (6%). Although they frequently had comorbid somatic disorder (mainly diseases of the nervous system and the musculoskeletal system and connective tissue), they had the lowest proportion of individuals receiving psychotherapy or rehabilitation.

### Individuals with Mixed States

We identified three groups with mixed states, two of which were composed mainly of young adults to whom temporary DP was granted. The minority of them, ´*mixed states to mixed states’* (*n* = 323), returned to work, studies, or unemployment. This was the youngest group, with an average age of 22 years, and individuals were primarily students or had several temporary work or unemployment periods before disability. The group had the highest proportion of individuals with an (F4*) “Neurotic, stress-related and somatoform disorders” diagnosis as well as of individuals receiving psychotherapy and without inpatient treatment. Most young adults, ‘*mixed states to temporary disability’ -group* (*n* = 1458), remained in DP during the follow-up period. They had the highest prevalence of only basic education (55%), psychotic disorders (38%), long inpatient experience (62%), and involuntary treatment (35%). Compared to other young adults, they had more frequently received rehabilitation and long hospital treatment but more rarely a short one (Table 1).

The members of the `*Mixed states to permanent disability’ -group* (n = 2,749) were mainly unemployed or their SES was unknown before disability (Table 1). The distinguishing characteristic of this group was the high proportion of pervasive and specific developmental disorders F8* (6%) as well as of substance-related diagnosis (7%), which was also a common comorbid psychiatric diagnosis (5%). In addition, individuals frequently had comorbid somatic disorders: diseases of the nervous system, the musculoskeletal system and connective tissue.

**Fig. 2:**
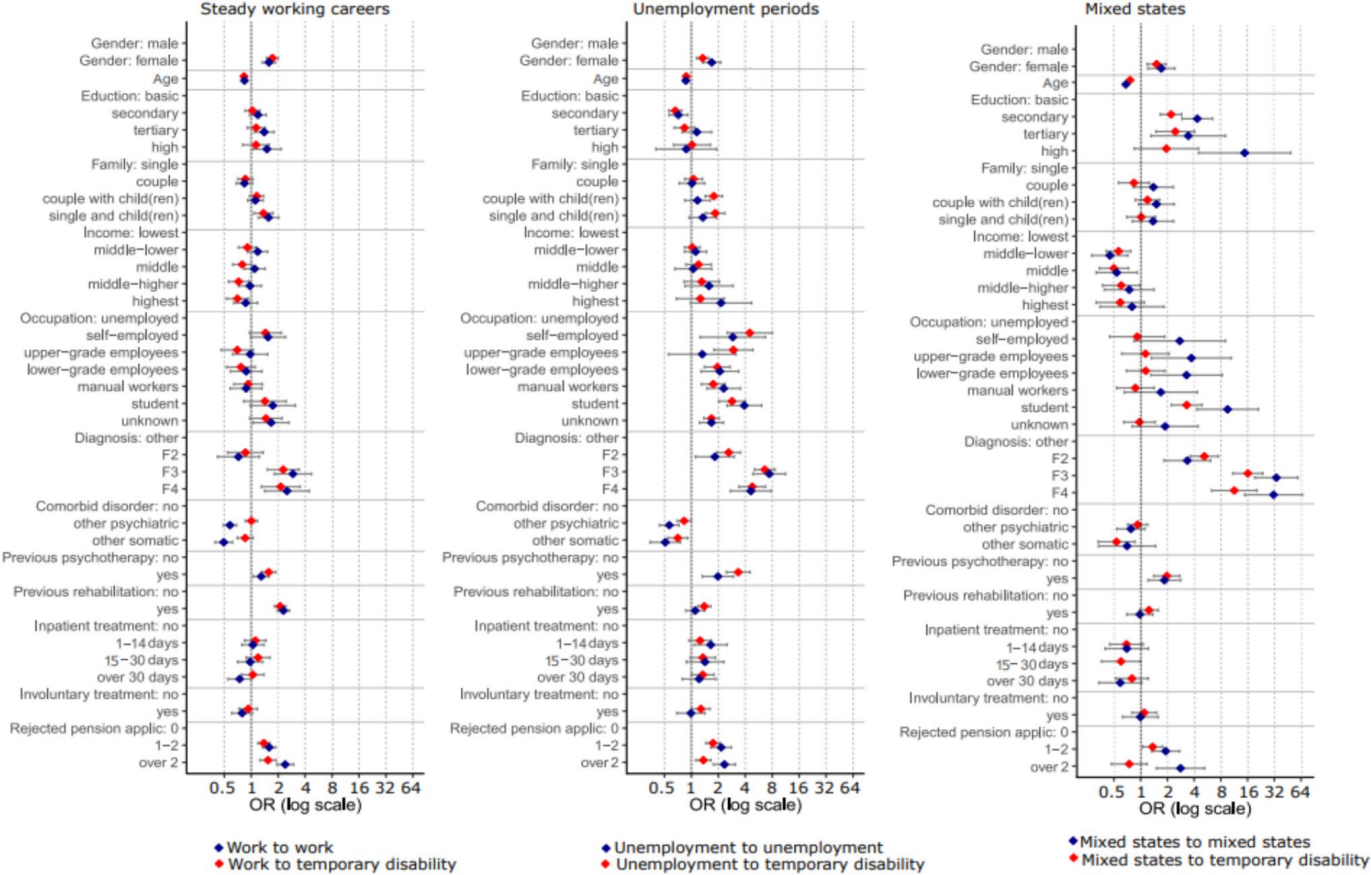
Differences between nine identified groups to and after a temporary and a permanent disability pension granted between 2010 and 2012. Associations of personal, socioeconomic, health and health care-related factors between people with **a** steady working careers, **b** unemployment periods and **c** mixed states in Finland by adjusted odds ratio (OR) and 95% confidence interval (CI) as a dot plot figure of final statistical model

Multinomial models complemented the results of cross-tabulation and were adjusted to ensure more reliable estimates. Based on the adjusted multinominal regression analysis (Fig. 2 and Supplementary material 2), individuals with temporary DP were younger and more frequently women compared to individuals granted permanent DP. When analysing individuals with a working career background, we detected that high education (OR 1.50; 95% CI 1.04–2.16) and having an affective disorder diagnosis (OR 2.92; 95% CI 1.81–4.72) or anxiety- or stress-related disorders F4 (OR 2.51; 95% CI 1.41–4.45) were highly associated with RTW. Furthermore, having an affective disorder diagnosis (OR 2.28; 95% CI 1.51–3.42) or anxiety- or stress-related disorders F4 (OR 2.14; 95% CI 1.30–3.51) were highly associated with remaining on long-term temporary DP compared to the individuals granted permanent DP.

Among individuals with an unemployment background, those who returned to unemployment had a higher risk of several rejected DP applications before their first DP compared to individuals with a permanent DP. Among individuals with mixed states those who were students (OR 9,54; 95%CI 4,29–21,20), with a high education (OR 14.84; 95%CI 4.50–49.00), a diagnosis of affective disorder (F3 OR 33.77; 95% CI 19.33–58.98) or anxiety or stress-related disorders (F4 OR 31.42; 95% CI 14.87–66.38) had higher probability of returning to work, studies or unemployment than in the case of individuals who were granted permanent DP.

## Discussion

Our study examined paths to and after disability pensions (DPs) due to mental disorders. Generally, individuals with temporary DPs appeared either to return to the same state they were in before pensioning (frequently to work or unemployment) or to remain in DP. Only a minority of the study population returned to work: 17% of individuals with temporary DP. The probability of return to work (RTW) appeared to be connected to a steady working career, high education, less severe mental disorders and to awarded rehabilitative psychotherapy or occupational rehabilitation.

Somewhat disturbingly, most young adults remained in DP during the three-year follow-up period, reflecting a high risk of never entering working life. This may justify paying special attention to this group. Not only severe mental disorders but also unemployment periods and a lack of occupational education appeared to be associated with prolonged work disability. This may be related to the average onset age of schizophrenia in young adulthood [39] as well as to poor mental health as a reason for dropping out of studies and other paths of opportunities [40]. Furthermore, young adults may lack the necessary coping strategies and social support networks. Additionally, the lack of work experience might make it more challenging for young adults to find flexible employment.

Based on our data, occupational rehabilitation and psychotherapy appeared not to be straightforwardly associated with the working ability of young adults, particularly those with severe mental disorders (F20-29). Our finding partially contradicts one recent review study, which found evidence that mental health treatment and youth-centred employment interventions may support young adults with mental disorders to obtain employment [41]. A possible explanation for this contradiction could be that some young adults with severe mental disorders are offered less effective treatment and interventions than others. In any case, it seems important to develop cost-effective and efficient treatment options and support to promote their work ability, health and independent living skills not only in social and health care but also in education systems [42]. We need policies and methods to minimize the risks accumulating in young adulthood, and to support young adults with severe mental health disorders to enter the active labour market or not to drop out of it, or to promote their education.

One effective approach to promoting employment among individuals with severe mental health disorders, is the Individual Placement and Support (IPS) model. This model integrates mental health rehabilitation with employment services. A Swedish study found that the IPS model outperformed traditional vocational rehabilitation in terms of employment rates, working hours, job tenures, and incomes [19]. Additionally, Finland’s recent National Mental Health Strategy 2030 highlighted the need for and supported the provision of IPS models for employment.

In our study, unemployment may be interpreted as an unrecognized work disability, or as a risk factor for exclusion from working life or for not to RTW after disability. Unemployment as an exclusion from working life accords with earlier studies, which have suggested that periods of unemployment and disability benefits may prolong absence from the labour market [20, 23, 24, 30]. This may also relate to a lack of suitable workplaces to return to.

In our study, long-term unemployed, or individuals with several unemployment periods, appeared also to be in the lowest-income households and to have comorbid somatic disorders and a substance-related diagnosis. From the point of view of the social exclusion theory, unemployment may generate a downward spiral towards poverty and social isolation, which in turn increases the risk of long-term unemployment [43]. Gallie and Paugman (2004) emphasised the significance of multiple deprivation for the experience of unemployment [43]. Long-term unemployment might also be connected to increased alcohol consumption and somatic diseases [44], which may have been partially reflected in our results.

Unemployment as an unrecognized work disability may relate to a contradiction between criteria for DP and an individual´s experienced work ability. In our study, long-term unemployment and several unemployment periods appeared to be related to a high number of rejected DP applications. This may indicate that individuals’ work disability was unrecognised by the DP system before their first granted DP and for individuals who returned to unemployment after it. This might be associated with the system-related challenges, assuming that people obtain sickness and disability benefits when unable to work due to illness, but for example long-term unemployed may receive unemployment allowance even when in fact permanently unable to work [45]*.* A Finnish study found that the majority of long-term unemployed with alcohol-related or affective disorders had impaired work ability. This impairment highlighted both the strengths and weaknesses of the health care system’s pathways*.* It would be important to train professionals working with long-term unemployed to be more perceptive in identifying mental health disorders, especially in the long-term unemployed with impaired work ability and somatic disorders [44]. Specifically allocated health care services for the unemployed would also recognize more efficiently whether rehabilitation, education, or earlier DP were more relevant options [5]. It would also be essential to offer more flexible job opportunities for those who cannot currently work at their full capacity. Altogether, better recognition of all these states, and of the requisite actions (pension, active rehabilitation, or continued unemployment allowance), would benefit both the system and the individuals affected.

Particular attention should be paid to long-term unemployment and repeated periods of unemployment, both of which may be detrimental for individuals, society, and social and health care systems. It might be necessary to consider procedures to prevent exclusion from working life by promoting rehabilitation and education. When prolonged unemployment is compounded with a low education level and comorbid mental and somatic disorders, occupational rehabilitation should occur at an early stage. It is also worth considering when individuals should be granted permanent DPs instead of temporary DP or unemployment benefit. It is possible to return to education or employment even from permanent DP. For individuals with no current prospects in the labour market and with insufficient abilities to study for more credentials, it might be more humane to grant a permanent DP instead of a temporary one or an unemployment benefit.

We identified two especially vulnerable groups: young adults with severe mental disorders and individuals with unemployment backgrounds. Based on an earlier study, low-income and poverty create significant barriers to accessing mental health services [17]. This might be an issue in Finland, because even though vocational rehabilitation is frequently free of charge, rehabilitative psychotherapy is only partially subsidized by the Finnish Centre for Pensions (FCP) [46]. Also challenges such as transportation, childcare responsibilities, lack of health insurance or funds, and inflexible work hours can prevent individuals from receiving necessary treatment and rehabilitation [16, 47]. Therefore, it is crucial to eliminate these obstacles to make mental health services more accessible [47].

The findings of our research provide insights for various population groups and paths to and after work disability, which might enhance recovery processes, in terms of increasing rates of RTW and prevention of exclusion from working life. Young adults with high education and middle-aged workers with steady working careers and affective disorders could have better possibilities to continue studies or RTW with active and effective rehabilitation, psychotherapy and work modifications.

## Scientific Implications

In this study we utilized a novel analysis method in this context. Cluster- or sequence analysis has potential over conventional statistical methods to identify temporal trends and paths. The results of our study suggest that clustering of individuals’ situations before and after DPs may provide a more accurate and realistic description of paths to and after disability.

## Strengths and Limitations

The main advantages of this study include the possibility to create a more actual and realistic description of individuals’ paths to long-term disability and their situations after DP, using cluster analysis together with more traditional statistical methods. We also used data based on reliable and high-quality register sources and comprehensive, national-level data. The study population included all new DPs granted due to mental disorder for 3 years in Finland; hence, we could systemically evaluate both the pensioning process and individuals’ situations after DP. Another strength is the broad range of sociodemographic factors which were linked at the individual level with longitudinal data. However, one limitation of the study is the relatively short 3-year follow-up period from the beginning of DP.

Work disability systems, benefits, terminologies and eligibilities vary between countries, limiting the generalizability and external validity of the results [2, 4], although the Finnish system corresponds with that in several other countries [19]. Despite these limitations, the study adds to our understanding of individuals’ paths to disability pension and their situations after DP.

## Concluding Remarks

The majority of the temporarily psychiatrically retired appear to remain on a DP after being granted one. Hence, there may exist vicious circles of disadvantage associated with prolonged unemployment, exclusion from working life, both mental and somatic disorders, low education and poverty. The rationale and usefulness of temporary DPs should perhaps be considered more carefully. When granted, they should be targeted to those with a potential to recover and should be associated with active vocational rehabilitation. There is also a need for policies and methods to minimise risks accumulating during an individual’s lifetime, as well as to prevent exclusion from working life, not only in social and health care but also in education systems. We need to promote rehabilitation and education for young adults with severe mental disorders.

## Supplementary Information

Below is the link to the electronic supplementary material.

Supplementary file1 (PDF 320 KB)

Supplementary file2 (PDF 141 KB)

## Data Availability

No datasets were generated or analysed during the current study.

## Abbreviations

FCP: The Finnish Centre for Pensions
DP: Disability pension
RTW: Return to work
SII: The National Social Insurance Institution
SF: Statistics Finland
tDP: Temporary disability pension
