# Supplementary material for: Return to Work or Not: The Paths to Psychiatric Disability and Back
Source: J Occup Rehabil. 2025 Aug 11;36(3):793–805. doi: 10.1007/s10926-025-10312-4 (PMC13364870; doi:10.1007/s10926-025-10312-4)
Supplement: Supplementary file 1 — Supplementary file1 (PDF 320 KB) [file 10926_2025_10312_MOESM1_ESM.pdf]

Differences between nine identified groups to and after a temporary and a permanent disability pension (DP) granted between 2010 and 2012. Associations of personal, socioeconomic, health and health care-related factors between people with a) steady working careers, b) unemployment periods and c) mixed states in Finland by adjusted odds ratio (OR), 95% confidence interval (CI) and significance. Reference groups: a) work to permanent DP b) unemployment to permanent DP c) mixed states to permanent DP

	**a) steady working careers**						**b) unemployment periods**						**c) mixed states**					
	**work to work**			**work to disability**			**unemployment to unemployment**			**unemployment to disability**			**mixed states to mixed states**			**mixed states to disability**		
	OR	95% CI	p	OR	95% CI	p	OR	95% CI	p	OR	95% CI	p	OR		p	OR		p
Female	1,577	(1,336–1,860)	<,001	1,724	(1,481–2,008)	<,001	1,686	(1,335–2,128)	<,001	1,335	(1,141–1,562)	<,001	1,707	(1,204–2,421)	0,003	1,514	(1,182–1,939)	0,001
Male	1																	
Age	0,84	(0,831–0,849)	<,001	0,831	(0,823–0,839)	<,001	0,867	(0,856–0,878)	<,001	0,875	(0,867–0,883)	<,001	0,680	(0,653–0,71)	<,001	0,759	(0,744–0,775)	<,001
Family situation Single	1																	
Couple	0,838	(0,681–1,031)	0,095	0,860	(0,711–1,039)	0,118	1,013	(0,731–1,406)	0,936	1,058	(0,842–1,33)	0,628	1,391	(0,827–2,341)	0,213	0,839	(0,563–1,250)	0,389
Single + children	1,111	(0,912–1,352)	0,295	1,151	(0,961–1,380)	0,126	1,170	(0,843–1,623)	0,348	1,779	(1,426–2,219)	<,001	1,506	(0,951–2,384)	0,081	1,193	(0,869–1,637)	0,275
Couple + children	1,561	(1,203–2,025)	0,001	1,369	(1,073–1,747)	0,011	1,345	(0,943–1,918)	0,101	1,844	(1,443–2,358)	<,001	1,379	(0,808–2,353)	0,239	1,014	(0,698–1,474)	0,942
Education level																		
Basic	1																	
Secondary	1,188	(0,959–1,470)	0,114	1,033	(0,855–1,249)	0,736	0,715	(0,561–0,91)	0,007	0,659	(0,559–0,778)	<,001	4,343	(2,915–6,47)	<,001	2,197	(1,665–2,898)	<,001
Tertiary	1,396	(1,084–1,797)	0,010	1,136	(0,903–1,427)	0,276	1,15	(0,788–1,678)	0,469	0,838	(0,642–1,093)	0,192	3,440	(1,308–9,049)	0,012	2,457	(1,484–4,07)	<,001
High	1,497	(1,039–2,156)	0,030	1,131	(0,804–1,59)	0,481	0,876	(0,401–1,915)	0,741	1,015	(0,634–1,624)	0,951	14,841	(4,495–48,998)	0,000	1,948	(0,847–4,481)	0,117
Disposable income of household																		
Lowest	1																	
Lower middle	1,18	(0,915–1,522)	0,201	0,904	(0,718–1,138)	0,39	1,114	(0,838–1,481)	0,458	1,023	(0,841–1,246)	0,818	0,450	(0,282–0,716)	0,001	0,564	(0,41–0,775)	0,000
Middle	1,091	(0,834–1,426)	0,525	0,793	(0,622–1,011)	0,061	1,05	(0,653–1,687)	0,841	1,202	(0,867–1,666)	0,269	0,537	(0,316–0,914)	0,022	0,499	(0,343–0,728)	0,000
Higher middle	0,968	(0,724–1,294)	0,826	0,723	(0,556–0,940)	0,016	1,572	(0,847–2,916)	0,152	1,306	(0,829–2,058)	0,249	0,744	(0,391–1,414)	0,366	0,604	(0,373–0,978)	0,041
Highest	0,867	(0,636–1,182)	0,367	0,701	(0,530–0,927)	0,013	2,14	(0,975–4,696)	0,058	1,259	(0,679–2,332)	0,465	0,799	(0,349–1,827)	0,595	0,589	(0,315–1,101)	0,097
Occupational situation																		
Unemployed	1			1			1			1			1			1		
Self employed	1,54	(0,981–2,42)	0,061	1,441	(0,958–2,167)	0,079	2,9	(1,252–6,717)	0,013	4,488	(2,524–7,98)	<,001	2,756	(0,843–9,014)	0,094	0,913	(0,448–1,858)	0,801
Upper grade employees	0,974	(0,621–1,528)	0,910	0,698	(0,461–1,056)	0,089	1,323	(0,556–3,148)	0,527	2,948	(1,788–4,863)	<,001	3,724	(1,322–10,486)	0,013	1,132	(0,614–2,088)	0,691
Lowe–grade employees	0,879	(0,587–1,318)	0,533	0,766	(0,531–1,106)	0,155	2,08	(1,282–3,376)	0,003	1,949	(1,402–2,71)	<,001	3,287	(1,302–8,3)	0,012	1,141	(0,693–1,878)	0,605
Manual worker	0,877	(0,583–1,318)	0,527	0,924	(0,641–1,332)	0,672	2,296	(1,493–3,531)	<,001	1,756	(1,295–2,381)	<,001	1,687	(0,655–4,348)	0,279	0,872	(0,536–1,419)	0,581
Student	1,74	(0,972–3,112)	0,062	1,424	(0,825–2,456)	0,204	3,901	(2,498–6,091)	<,001	2,849	(2,026–4,006)	<,001	9,541	(4,294–21,198)	0,000	3,299	(2,218–4,909)	<,001

Unknown	1,668	(1,054–2,638)	0,029	1,46	(0,963–2,212)	0,075	1,675	(1,231–2,28)	0,001	1,675	(1,376–2,041)	<,001	1,888	(0,804–4,434)	0,145	0,969	(0,645–1,457)	0,880
Diagnosis																		
Psychotic disorders	0,72	(0,421–1,234)	0,232	0,861	(0,546–1,359)	0,52	1,831	(1,111–3,019)	0,018	2,612	(1,929–3,536)	<,001	3,355	(1,834–6,135)	<,001	5,214	(3,622–7,505)	<,001
Affective disorders	2,92	(1,808–4,716)	<,001	2,277	(1,514–3,424)	<,001	7,414	(4,876–11,272)	<,001	6,605	(5,069–8,606)	<,001	33,767	(19,332–58,983)	<,001	16,126	(10,956–23,734)	<,001
Neurotic disorders	2,507	(1,413–4,45)	0,002	2,135	(1,297–3,514)	0,003	4,62	(2,731–7,817)	<,001	4,779	(3,402–6,715)	<,001	31,415	(14,867–66,381)	<,001	11,335	(6,285–20,441)	<,001
Other	1			1			1			1			1			1		
Comorbidity																		
No	1																	
Psychiatric	0,578	(0,489–0,684)	<,001	1,003	(0,858–1,173)	0,967	0,566	(0,44–0,727)	<,001	0,83	(0,694–0,991)	0,04	0,777	(0,54–1,118)	0,174	0,924	(0,714–1,196)	0,548
Somatic	0,493	(0,394–0,618)	<,001	0,855	(0,699–1,045)	0,126	0,511	(0,346–0,754)	0,001	0,705	(0,547–0,91)	0,007	0,704	(0,336–1,474)	0,352	0,535	(0,332–0,862)	0,010
Psychotherapy																		
Yes	1,293	(1,061–1,575)	0,011	1,566	(1,302–1,882)	<,001	1,981	(1,324–2,964)	0,001	3,339	(2,461–4,531)	<,001	1,852	(1,21–2,833)	0,005	1,970	(1,405–2,763)	0,000
No	1																	
Rehabilitation																		
Yes	2,291	(1,96–2,678)	<,001	2,104	(1,82–2,432)	<,001	1,102	(0,866–1,403)	0,43	1,393	(1,178–1,648)	<,001	0,979	(0,697–1,374)	0,903	1,240	(0,976–1,575)	0,078
No	1			1			1			1			1			1		
Inpatient treatment																		
No	1			1			1			1			1			1		
1–13 days	1,044	(0,785–1,39)	0,766	1,11	(0,843–1,462)	0,455	1,643	(1,079–2,501)	0,021	1,25	(0,94–1,661)	0,125	0,698	(0,398–1,223)	0,209	0,692	(0,448–1,069)	0,097
14–30 days	0,973	(0,705–1,343)	0,869	1,193	(0,878–1,619)	0,259	1,419	(0,877–2,298)	0,154	1,343	(0,971–1,859)	0,075	0,239	(0,111–0,515)	<,001	0,600	(0,363–0,993)	0,047
Over 30 days	0,742	(0,548–1,004)	0,053	1,042	(0,782–1,388)	0,778	1,22	(0,791–1,88)	0,368	1,34	(1,004–1,789)	0,047	0,589	(0,339–1,024)	0,061	0,797	(0,521–1,218)	0,295
Involuntary treatment																		
Yes	0,791	(0,606–1,032)	0,084	0,925	(0,732–1,17)	0,516	0,987	(0,69–1,414)	0,945	1,278	(1,012–1,612)	0,039	0,994	(0,627–1,575)	0,979	1,096	(0,796–1,507)	0,575
No	1																	
Rejected DP applications																		
No	1																	
1 or 2	1,587	(1,346–1,872)	<,001	1,382	(1,189–1,608)	<,001	2,15	(1,652–2,798)	<,001	1,741	(1,455–2,084)	<,001	1,915	(1,335–2,745)	<,001	1,366	(1,057–1,766)	0,017
3 and over	2,396	(1,933–2,971)	<,001	1,538	(1,252–1,891)	<,001	2,34	(1,764–3,103)	<,001	1,361	(1,121–1,652)	0,002	2,811	(1,506–5,247)	0,001	0,743	(0,469–1,179)	0,208
Goodness–of–fit																		
Pearson chi2	15380,25						12460,580						6195,784					

Nagelkerke	0,507	0,487	0,763
