# Supplementary figures and images for: Return to Work or Not: The Paths to Psychiatric Disability and Back
Source: J Occup Rehabil. 2025 Aug 11;36(3):793–805. doi: 10.1007/s10926-025-10312-4 (PMC13364870; doi:10.1007/s10926-025-10312-4)

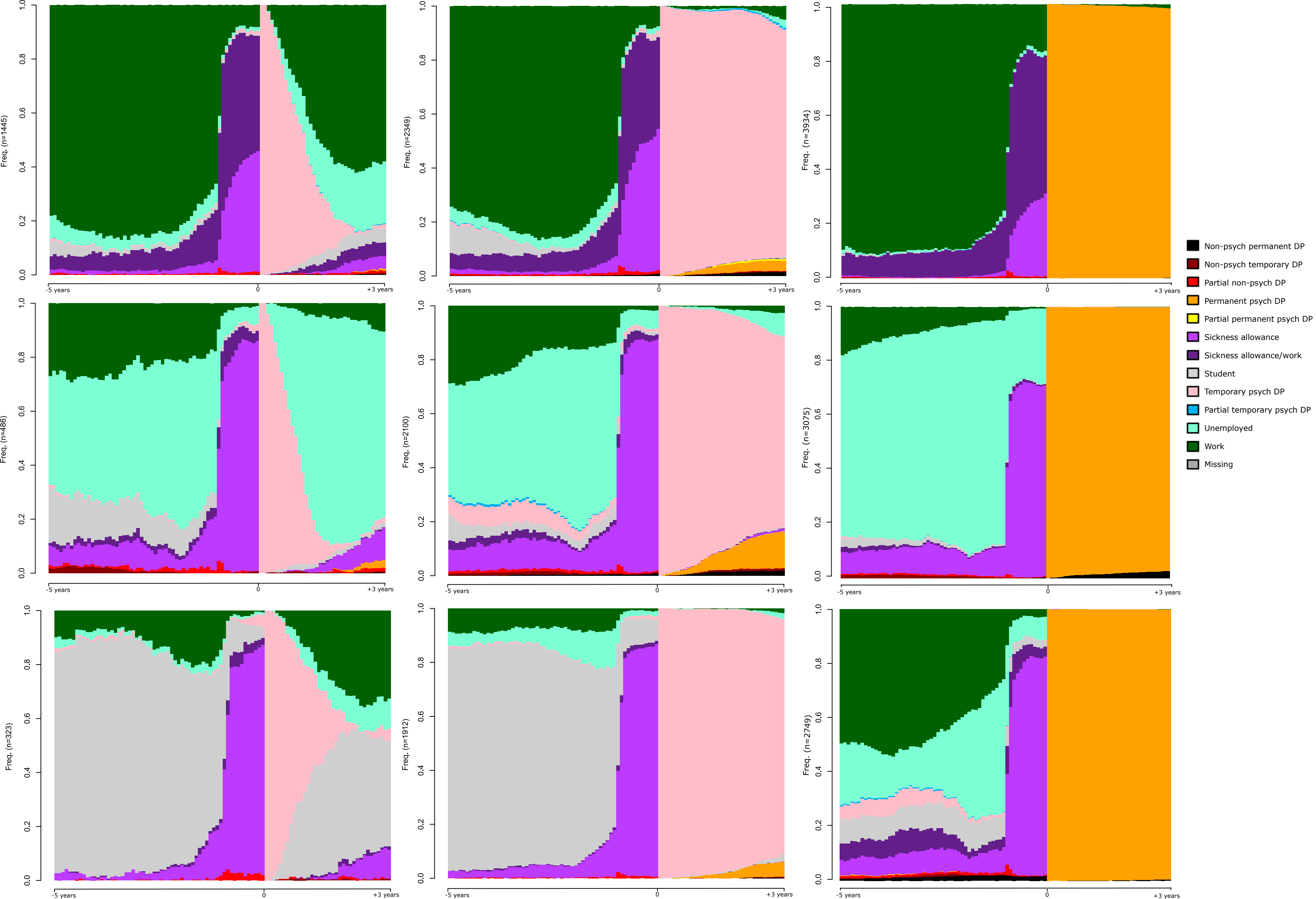

Supplement: Supplementary file 2 — Supplementary file2 (PDF 141 KB) [file 10926_2025_10312_MOESM2_ESM.pdf]
